# The Burden and Characteristics of Post-COVID-19 Conditions Among Laboratory-Confirmed Delta and Omicron COVID-19 Cases: A Preliminary Study From Maharashtra, India

**DOI:** 10.7759/cureus.44888

**Published:** 2023-09-08

**Authors:** Rajesh P Karyakarte, Rashmita Das, Mansi V Rajmane, Sonali Dudhate, Jeanne Agarasen, Praveena Pillai, Priyanka M Chandankhede, Rutika S Labhshetwar, Yogita Gadiyal, Preeti P Kulkarni, Safanah Nizarudeen, Savita Mukade, Suvarna Joshi

**Affiliations:** 1 Microbiology, Byramjee Jeejeebhoy (BJ) Government Medical College and Sassoon General Hospitals, Pune, IND; 2 Microbiology, Byramjee Jeejeebhoy Government Medical College (BJGMC), Pune, IND

**Keywords:** sars-cov-2, covid-19, long haulers, post-acute sequelae of sars-cov-2 (pasc), post-covid-19 condition (pcc), long covid

## Abstract

Background: Post-COVID-19 conditions (PCC) have emerged as a significant global health concern due to their potential impact on patients’ quality of life and healthcare resources. The present study aims to understand the burden and characteristics of PCC in Maharashtra, India, and compares its prevalence among cases infected with Delta and Omicron variants.

Material and methods: A retrospective observational study included 617 laboratory-confirmed Delta and Omicron variant cases. These cases were telephonically followed up to document persistent COVID-19 symptoms using a questionnaire based on the Post-COVID-19 Clinical Form from the Global COVID-19 Clinical Platform of the World Health Organization (WHO), and the results were analyzed.

Results: Out of 617 laboratory-confirmed COVID-19 cases, 82.97% and 17.03% were Omicron and Delta cases, respectively. The mean follow-up period for Delta and Omicron cases was 78.05 and 21.56 weeks, respectively. A total of 40 (6.48%) cases reported persistent symptoms at follow-up, with a higher prevalence among those infected with the Delta variant (12.38%) compared to the Omicron variant (5.27%). The most common long COVID symptoms reported were malaise (25%), dyspnea (20%), post-exertional fatigue (17.5%), joint pain (15%), and frequent episodes of cough and cold (15%). Additionally, 1.94% of participants developed a new medical condition following COVID-19 infection, most commonly hypertension (25%), lung fibrosis (16.67%), and asthma (8.33%). Factors such as more than five acute symptoms, a moderate to severe disease, the need for hospitalization, and hospitalization for more than five days were significantly associated with PCC.

Conclusion: Long COVID results in extended disability and illness. The varying impacts of different COVID-19 variants highlight the complex nature of post-COVID-19 complications. Our findings highlight the need for strategic planning of healthcare resources to ensure optimal response and preparedness to manage the burden of PCC.

## Introduction

It has been three years since the declaration of the COVID-19 pandemic, and several variants of concern/interest (VoC/VoI) have been identified, which differ in transmissibility, disease course, disease severity, and response to treatment and vaccines [1]. Extensive studies have been conducted to gather evidence and understanding of the acute symptoms of the disease caused by various SARS-CoV-2 variants. It causes diseases ranging from asymptomatic or mild respiratory disease to life-threatening diseases leading to death. As most patients return to baseline within two to four weeks of an acute infection, a proportion of people have reported ongoing health issues [2]. This long-term sequela is commonly referred to as long COVID or post-COVID-19 condition (PCC) [3].

The concept of post-COVID-19 conditions was first described by the Centers for Disease Control and Prevention (CDC) in November 2020, a few months after the onset of the pandemic, when otherwise healthy individuals experienced persistent symptoms weeks and months after recovery. These patients called themselves “long haulers” and were labeled PCC in February 2021 [4]. Since then, the definition of these conditions has been evolving. The CDC defines PCC as a wide range of new, returning, or ongoing health problems that people experience at least four or more weeks after being infected with the SARS-CoV-2 virus. This term is used as an umbrella term for a wide range of physical, social, and psychological health consequences experienced by patients [5]. Similarly, the World Health Organization (WHO) defines it as the continuation or development of new symptoms three months after the initial SARS-CoV-2 infection, lasting at least two months with no other explanation [3]. While long COVID has recently been recognized as a distinct condition, several ongoing research aim to understand its causes, risk factors, and potential treatments. The CDC’s “Innovative Support for Patients with SARS-CoV-2 Infections” (INSPIRE) and the National Institutes of Health’s (NIH) “Researching COVID to Enhance Recovery” (RECOVER) initiatives are helping in a better understanding of the long-term effects of COVID-19 infection [5]. Although exact numbers are uncertain, studies have shown that around 10%-20% of people with SARS-CoV-2 infection may develop long COVID symptoms. It has been estimated that more than 17 million people in the WHO European Region may have experienced these symptoms during the first two years of the pandemic [3].

While many studies are ongoing in the West, very few studies are available from India assessing the burden of PCC. The Ministry of Health and Family Welfare, Government of India, has also developed a comprehensive guideline for managing post-COVID-19 sequelae [6]. With around 45 million cases of SARS-CoV-2 infection reported from India to date [7], long COVID can become a significant public health concern. Therefore, the present study was conducted to measure the burden and understand the characteristics of post-COVID-19 conditions in Maharashtra. The study also compares the incidence and difference of PCC in cases infected with the Delta and Omicron variants.

## Materials and methods

A retrospective observational study was conducted at the Department of Microbiology, Byramjee Jeejeebhoy Government Medical College and Sassoon General Hospitals (BJGMC and SGH), Pune, Maharashtra. The study’s objective was to estimate the percentage of the study population reporting long COVID symptoms by following up a group of COVID-19 reverse transcriptase polymerase chain reaction (RT-PCR)-positive individuals whose nasopharyngeal samples were sequenced to obtain available SARS-CoV-2 variant information.

Data collection

BJGMC and SGH, Pune, had previously conducted clinical studies to characterize the symptoms of patients infected with Delta [8] and Omicron variants [9,10]. The database of these patients was used in the present study. The baseline data were extracted from the database, including their demographic details, symptoms that developed during acute COVID-19 infection, treatment received, and their COVID-19 vaccination history at the time of infection.

BJGMC, Pune, is Maharashtra’s coordinating center for SARS-CoV-2 whole genome sequencing. Therefore, SARS-CoV-2 variant information for each participant was readily available and was used to classify them into cases either infected with Delta or Omicron variants. The genome sequencing activity was carried out at Byramjee Jeejeebhoy Government Medical College, Pune, National Institute of Virology, Pune, and the Indian Institute of Science Education and Research, Pune, under the Indian SARS-CoV-2 Genomics Consortium (INSACOG). This variant data was used to compare the incidence and difference in post-COVID-19 symptoms in participants infected during the Delta and Omicron waves.

**Figure 1:**
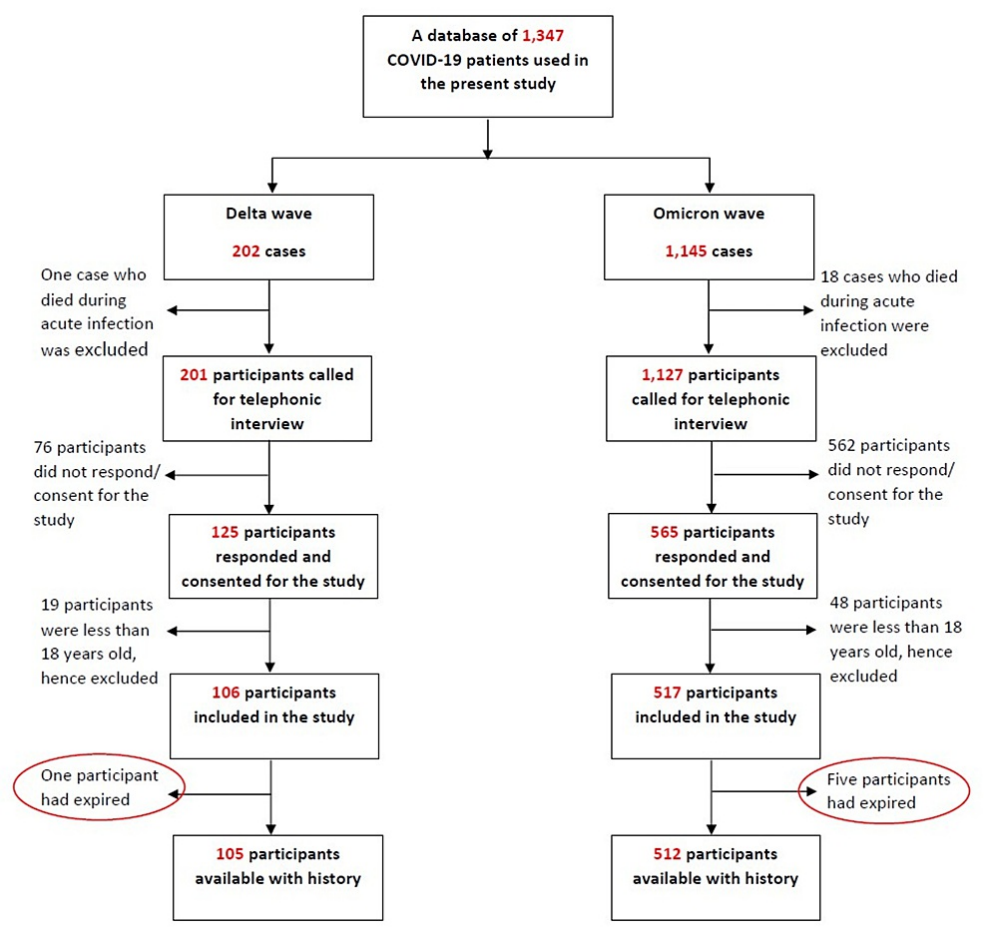
Flowchart showing the selection of participants for the study COVID-19: coronavirus disease 2019

The participants were followed up telephonically. Verbal consent was obtained from each participant. A detailed interview was conducted to document the presence of any persistent symptoms such as cough, loss of smell and taste, fatigue, cognitive dysfunction, sleep disorder, and weight loss. The questionnaire for the interview was based on the Post-COVID-19 Clinical Form from the Global COVID-19 Clinical Platform of the WHO [11]. Participants under 18 years of age and those who could not be contacted or did not consent to the study were excluded (Figure 1).

Statistical analysis

All data collected during the study were entered into Microsoft Excel (Microsoft Corp., Redmond, WA, USA) and analyzed using Minitab® version 21.3.1 (State College, PA, USA) statistical software. Categorical variables were represented as percentages, and continuous variables were presented as mean (± standard error of mean (SEM)) or median (interquartile range (IQR)). For comparison between variables during the two waves, categorical variables were compared using the Fisher exact or Chi-square test, and continuous variables were compared using the independent sample Student’s t-test. A p-value of ≤0.05 was considered statistically significant.

## Results

Out of the 1,347 laboratory-confirmed COVID-19 cases, 1,329 participants were contacted, of which 690 (51.92%) participants responded. Sixty-seven (9.71%) participants under the age of 18 years and six (0.87%) participants who were reported dead were excluded from the study. Therefore, 617 out of 690 participants, i.e., 89.42%, were eligible and included in the study.

SARS-CoV-2 lineage distribution among 617 RT-PCR-positive samples

Table 1 shows the variant distribution of 617 RT-PCR cases included in the study, of which 512 (82.97%) cases were infected with the Omicron variant and 105 (17.02%) were infected with the Delta variant. Among the Omicron variant, BA.2.75* (57.81%) was the most common Omicron sub-lineage, followed by the XBB* recombinant variant (12.30%). Similarly, B.1.617.2 (64.76%) was the predominant Delta lineage among the Delta variant, followed by the AY.4 (17.14%) variant.

**Table 1:** Variant distribution of 617 RT-PCR-positive cases included in the study RT-PCR: reverse transcriptase polymerase chain reaction, WHO: World Health Organization

WHO nomenclature	Date of sample collection	Nextclade	Pangolin lineages	Numbers (percentage)	
Delta	July 1, 2021, to September 1, 2021	21A	B.1.617.2	68 (11.02%)	105 (17.02%)
		21J	AY.4	18 (2.92%)	
		21J	AY.12	10 (1.62%)	
		21J	AY.10	3 (0.49%)	
		21J	AY.16	3 (0.49%)	
		21J	AY.5	1 (0.16%)	
		21J	AY.11	1 (0.16%)	
		21J	AY.25	1 (0.16%)	
Omicron	May 19, 2022, to November 19, 2022	22D	BA.2.75*	296 (47.97%)	512 (82.97%)
		22F	XBB*	63 (10.21%)	
		21L	BA.2.74	51 (8.27%)	
		21L	BA.2.38*	41 (6.65%)	
		21L	BA.2.76	29 (4.70%)	
		22B	BA.5*	20 (3.24%)	
		21L	BA.2.10*	9 (1.46%)	
		21L	BA.2.3.20	1 (0.16%)	
		22E	BQ.1	1 (0.16%)	
			XAR	1 (0.16%)	
		Total		617 (100%)	

Demographic profile of the participants enrolled in the study

The mean age of the study population was 42.92 ± 0.67 years, with 270 (43.76%) females and 347 (56.24%) males. Table 2 describes the geographical distribution of the participants enrolled in the study. Most participants (82.17%) belonged to the Pune district, followed by Thane (5.51%) and Kolhapur (4.54%) districts.

**Table 2:** Geographical distribution of the participants included in the study

Area of residence		Numbers (percentage)
Within Maharashtra	Ahmednagar	3 (0.49%)
	Akola	3 (0.49%)
	Alibag	7 (1.13%)
	Amravati	2 (0.32%)
	Aurangabad	1 (0.16%)
	Baramati	2 (0.32%)
	Beed	4 (0.65%)
	Buldhana	1 (0.16%)
	Jalna	2 (0.32%)
	Kolhapur	28 (4.54%)
	Mumbai	3 (0.49%)
	Nagpur	3 (0.49%)
	Nashik	1 (0.16%)
	Parbhani	1 (0.16%)
	Pune	507 (82.17%)
	Sangli	1 (0.16%)
	Satara	8 (1.30%)
	Solapur	7 (1.13%)
	Thane	34 (5.51%)
Outside Maharashtra	Goa	3 (0.49%)
	Silvassa	1 (0.16%)
	Total	617 (100%)

The demographic characteristics of the participants infected during the Delta and Omicron waves are described in Table 3. The mean age of participants infected during the Delta and Omicron waves was 39.84 ± 1.47 and 43.56 ± 0.75. Males were more affected than females during both waves. Among the 270 females, 15 (5.56%) were identified as antenatal cases. Out of the 617 participants, 76 (12.32%) reported the presence of a pre-existing comorbid condition, of which the majority (56.58%) had hypertension, followed by diabetes mellitus (43.42%), asthma/bronchitis (6.58%), renal disease, malignancy, and coronary artery disease (3.95% each). Seventy-one (11.51%) participants reported a history of previous COVID-19 infection. Of these 617 participants, 552 (89.47%) had received at least one dose of vaccine at the time of acute infection, while 60 (9.72%) were unvaccinated. Notably, the proportion of vaccinated participants during the Delta wave was lower (55.24%) than those infected during the Omicron wave. The observed differences in the percentages of vaccinated and unvaccinated individuals during the waves may have resulted from the vaccination policies implemented during the pandemic [12].

**Table 3:** Demographic profile of the 617 participants included in the study COVID-19: coronavirus disease 2019

	Number of participants infected during the Delta wave (%)	Number of participants infected during the Omicron wave (%)	Grand total (%)	p-value
1. Gender-wise distribution				0.032
Male	69 (65.71%)	278 (54.30%)	347 (56.24%)	
Female	36 (34.29%)	234 (45.70%)	270 (43.76%)	
2. Mean age ± standard error of mean				<0.001
	39.84 ± 1.47	43.56 ± 0.75		
3. Age-wise distribution				0.306
18-19	3 (2.86%)	11 (2.15%)	14 (2.27%)	
20-29	29 (27.62%)	109 (21.29%)	138 (22.37%)	
30-39	25 (23.81%)	138 (26.95%)	163 (26.42%)	
40-49	21 (20%)	73 (14.26%)	94 (15.23%)	
50-59	12 (11.43%)	76 (14.84%)	88 (14.26%)	
60-69	9 (8.57%)	53 (10.35%)	62 (10.05%)	
70-79	6 (5.71%)	37 (7.23%)	43 (6.97%)	
80-89	0 (0%)	15 (2.93%)	15 (2.43%)	
4. Presence of pre-existing comorbid conditions				0.011
Comorbid condition absent	83 (79.05%)	458 (89.45%)	541 (87.68%)	
Presence of one condition	17 (16.19%)	44 (8.59%)	61 (9.89%)	
Presence of two or more conditions	5 (4.76%)	10 (1.95%)	15 (2.43%)	
5. History of previous COVID-19 infection				<0.001
No	105 (100%)	441 (86.13%)	546 (88.49%)	
Yes	0 (0%)	71 (13.87%)	71 (11.51%)	
6. Vaccination status at the time of acute infection				<0.001
Vaccinated with at least one dose	58 (55.24%)	499 (97.46%)	557 (90.28%)	
Single dose	35 (60.34%)	13 (2.61%)	48 (8.62%)	
Two doses	23 (39.66%)	362 (72.54%)	385 (69.12%)	
Booster dose	0 (0%)	124 (24.85%)	124 (22.26%)	
Not vaccinated	47 (44.76%)	13 (2.54%)	60 (9.72%)	

Clinical profile of the participants enrolled in the study

Table 4 describes the clinical characteristics of the participants at the time of acute COVID-19 infection. Fifty-six (9.08%) participants had an asymptomatic infection, and 561 (90.92%) had a symptomatic disease. The most common symptoms included fever (68.56%), followed by cough (41.98%), rhinorrhea (38.09%), fatigue (17.67%), and myalgia (14.91%). Symptoms such as fever, rhinorrhea, and fatigue were significantly higher among Omicron-infected participants, while loss of taste and smell was significantly high among the participants infected with the Delta variant. A higher proportion of participants with Delta infection were institutionally quarantined/hospitalized. This difference in proportion is again attributed to a change in quarantine policies during both waves [13,14]. Out of the 144 hospitalized cases, 24 (16.67%) needed oxygen therapy, six (4.17%) required antiviral/immunosuppressive therapy, nine (6.25%) required both oxygen and antiviral therapy, and 105 (72.92%) recovered with conservative treatment.

**Table 4:** Comparison of the clinical profile of the 617 participants during Delta and Omicron waves

	Number of participants infected during the Delta wave	Number of participants infected during the Omicron wave	Grand total	p-value
1. Symptom status at the time of acute infection				<0.001
Asymptomatic	20 (19.05%)	36 (7.03%)	56 (9.08%)	
Symptomatic	85 (80.95%)	476 (92.97%)	561 (90.92%)	
2. Initial presenting symptoms				
Fever	48 (45.71%)	375 (73.24%)	423 (68.56%)	<0.001
Cough	36 (34.29%)	223 (43.55%)	259 (41.98%)	0.080
Rhinorrhea	28 (26.67%)	207 (40.43%)	235 (38.09%)	0.008
Fatigue/weakness	9 (8.57%)	100 (19.53%)	109 (17.67%)	0.007
Myalgia	0 (0%)	92 (17.97%)	92 (14.91%)	-
Headache	6 (5.71%)	58 (11.33%)	64 (10.37%)	0.086
Sore throat	2 (1.90%)	31 (6.05%)	33 (5.35%)	0.085
Breathlessness	4 (3.81%)	21 (4.10%)	25 (4.05%)	0.890
Loss of smell	11 (10.48%)	4 (0.77%)	15 (2.43%)	<0.001
Diarrhea	0 (0%)	14 (2.73%)	14 (2.27%)	-
Loss of taste	9 (8.57%)	3 (0.58%)	12 (1.94%)	<0.001
Vomiting	1 (0.95%)	10 (1.95%)	11 (1.78%)	0.480
Chest pain	0 (0%)	3 (0.59%)	3 (0.49%)	-
Abdominal pain	1 (0.95%)	02 (0.39%)	3 (0.49%)	-
Skin rash	0 (0%)	01 (0.20%)	1 (0.16%)	-
3. Type of quarantine				<0.0001
Home quarantine	55 (52.38%)	418 (81.64%)	473 (76.66%)	
Institutional quarantine/required hospitalization	50 (47.62%)	94 (18.36%)	144 (23.34%)	
4. Treatment				<0.0001
No treatment taken	19 (18.10%)	16 (3.13%)	35 (5.67%)	
Needed conservative treatment	78 (74.29%)	465 (90.82%)	543 (88.01%)	
Needed oxygen/antiviral agents/steroids	8 (7.62%)	31 (6.05%)	39 (6.32%)	
Needed supplemental oxygen	6 (5.71%)	18 (3.52%)	24 (3.89%)	
*Low-flow oxygen	5 (83.33%)	15 (83.33%)	20 (83.33%)	
*Intubation	1 (16.67%)	3 (16.67%)	4 (16.67%)	
Needed antiviral agents/steroids or immunomodulatory drugs	0 (0%)	6 (1.17%)	6 (0.97%)	
Needed both oxygen therapy and antiviral agents/steroid or immunomodulatory drugs	2 (1.90%)	7 (1.37%)	9 (1.46%)	

Presence of self-reported post-COVID-19 symptoms among 617 participants

The mean duration of follow-up was 546.34 ± 0.72 days (~78.05 weeks) and 150.92 ± 2.22 days (~21.56 weeks) for participants infected with Delta and Omicron variants, respectively. Table 5 describes the characteristics of post-COVID-19 symptoms among the participants. Forty (6.48%) participants reported persistent symptoms during follow-up. Thirteen out of 92 (12.38%) participants infected during the Delta wave reported persistent symptoms compared to 27 out of 485 (5.27%) participants infected during the Omicron wave. Generalized weakness (25%) was the most common symptom reported, followed by shortness of breath (20%), post-exertional fatigue (17.5%), and joint pain (15%). Shortness of breath and joint pain were significantly higher among participants infected with the Delta variant. Twelve (1.94%) participants reported the development of a new disease after COVID-19 infection, of which hypertension (25%) was the common condition, followed by lung fibrosis (16.67%) and asthma (8.33%).

**Table 5:** Comparison of post-COVID-19 symptoms among the 617 participants during the Delta and Omicron waves COVID-19: coronavirus disease 2019

	Number of participants infected during the Delta wave	Number of participants infected during the Omicron wave	Grand total	p-value
1. Presence of self-reported post-COVID-19 symptoms during follow-up				0.007
Symptoms present	13 (12.38%)	27 (5.27%)	40 (6.48%)	
Symptoms absent	92 (87.62%)	485 (94.73%)	577 (93.52%)	
2. Most common post-COVID-19 symptoms reported				
General symptoms				
Malaise	3 (23.08%)	7 (26.92%)	10 (25%)	0.386
Weight gain	2 (15.38%)	0 (0%)	2 (5%)	-
Weight loss	0 (0%)	2 (7.69%)	2 (5%)	-
Persistent headache	0 (0%)	1 (3.85%)	1 (2.5%)	-
Respiratory symptoms				
Shortness of breath	5 (38.46%)	3 (11.11%)	8 (20%)	0.005
Frequent episodes of cold and cough/infections	3 (23.08%)	3 (11.11%)	6 (15%)	0.065
Persistent cough	0 (0%)	1 (3.85%)	1 (2.5%)	-
Neurological symptoms				
Anxiety	0 (0%)	2 (7.69%)	2 (5%)	-
Reduced smell	1 (7.69%)	1 (3.85%)	2 (5%)	0.312
Sleep disorder	0 (0%)	2 (7.69%)	2 (5%)	-
Reduced taste	0 (0%)	1 (3.85%)	1 (2.5%)	-
Reduced vision	0 (0%)	1 (3.85%)	1 (2.5%)	-
Increased sweating	1 (7.69%)	0 (0%)	1 (2.5%)	-
Gastrointestinal symptoms				
Constipation	1 (7.69%)	0 (0%)	1 (2.5%)	-
Musculoskeletal symptoms				
Post-exertional fatigue	3 (23.08%)	4 (15.38%)	7 (17.5%)	0.099
Joint pain	4 (30.77%)	2 (7.69%)	6 (15%)	0.009
Myalgia	4 (30.77%)	0 (0%)	4 (10%)	-
Dermatological symptoms				
Body rash (on and off)	0 (0%)	1 (3.85%)	1 (2.5%)	-
3. Any newly diagnosed condition after a COVID-19 infection				0.131
Yes	4 (3.81%)	8 (1.56%)	12 (1.94%)	
No	101 (96.19%)	504 (98.44%)	605 (98.06%)	
4. Common conditions diagnosed after COVID-19 infection				
Hypertension	1 (25%)	2 (25%)	3 (25%)	0.429
Lung fibrosis	1 (25%)	1 (12.5%)	2 (16.67%)	0.312
Asthma	0 (0%)	1 (12.5%)	1 (8.33%)	-
Malignancy	0 (0%)	1 (12.5%)	1 (0.83%)	-
Tuberculosis	0 (0%)	1 (12.5%)	1 (0.83%)	-
Rheumatoid arthritis	0 (0%)	1 (12.5%)	1 (0.83%)	-
Hypothyroidism	0 (0%)	1 (12.5%)	1 (0.83%)	-
Cataract	1 (25%)	0 (0%)	1 (0.83%)	-
Diabetes mellitus	1 (25%)	0 (0%)	1 (0.83%)	-

Excluding the 617 participants enrolled for the study, six participants (one infected with the Delta variant and five infected with the Omicron variant) were alive at the time of the first interview for acute infection. However, they succumbed to the disease following COVID-19 infection. All six individuals were elderly, aged above 60 years. While the cause of death in the three cases was lung fibrosis, pneumonia, and sudden myocardial infarction, the cause of death for the other three cases was not available.

Risk factors for the development of self-reported post-COVID-19 symptoms

**Figure 2:**
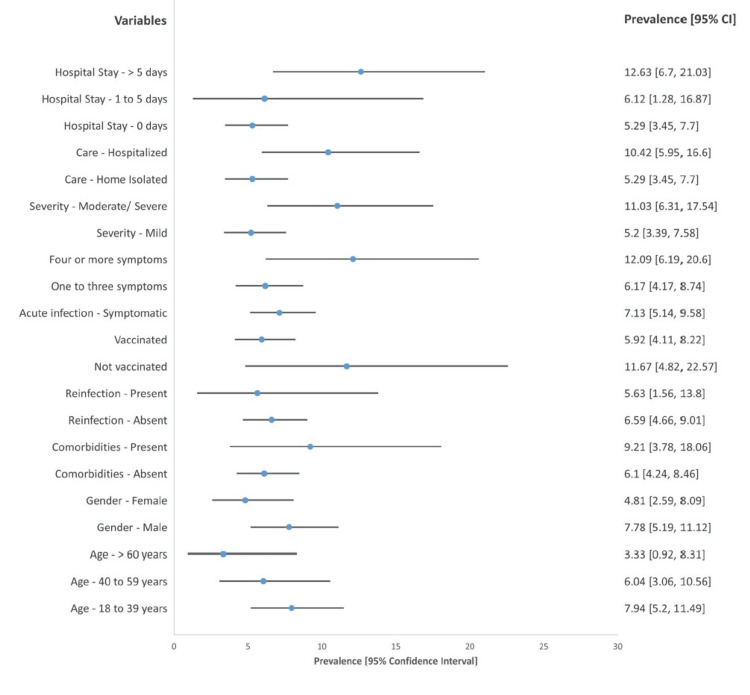
Forest plot demonstrating the prevalence of post-COVID-19 conditions among different variable groups COVID-19: coronavirus disease 2019

Participants with more than five acute COVID-19 symptoms, with moderate to severe disease, who needed hospitalization, and who were hospitalized for more than five days were significantly associated with post-COVID-19 symptoms. While other factors such as age, gender, presence or absence of comorbidities, prior COVID-19 infection, and vaccination status at the time of acute infection showed different prevalence across categories, the differences were not statistically significant (Table 6, Figure 2).

**Table 6:** Risk factors for the development of self-reported post-COVID-19 symptoms COVID-19: coronavirus disease 2019

Variables	Post-COVID-19 symptoms present (%)	Post-COVID-19 symptoms absent (%)	p-value
1. Age			0.210
18-39 years	25 (7.94%)	290 (92.06%)	
40-59 years	11 (6.04%)	171 (93.96%)	
≥60 years	4 (3.33%)	116 (96.67%)	
2. Gender			0.187
Male	27 (7.78%)	320 (92.22%)	
Female	13 (4.81%)	257 (95.82%)	
3. Presence of comorbidities			0.434
Comorbid conditions absent	33 (6.10%)	508 (93.90%)	
Comorbid conditions present	7 (9.21%)	69 (90.79%)	
4. History of re-infection			0.958
Absent	36 (6.59%)	510 (93.41%)	
Present	4 (5.63%)	67 (94.37%)	
5. Vaccination status at the time of acute infection			0.150
Not vaccinated	7 (11.67%)	53 (88.33%)	
Vaccinated	33 (5.92%)	524 (94.08%)	
6. Symptom status at the time of acute infection			0.075
Asymptomatic	0 (0%)	56 (100%)	
Symptomatic	40 (7.13%)	521 (92.87%)	
7. Number of symptoms present at the time of acute infection			0.013
No symptoms present	0 (0%)	56 (100%)	
One to three symptoms present	29 (6.17%)	441 (93.83%)	
More than four symptoms present	11 (12.09%)	80 (87.91%)	
8. Type of isolation			0.046
Home isolation	25 (5.29%)	448 (94.71%)	
Hospitalization	15 (10.42%)	129 (89.58%)	
9. Severity of acute infection			0.025
Mild disease	25 (5.20%)	456 (94.80%)	
Moderate/severe disease	15 (11.03%)	121 (88.97%)	
10. Number of days of hospitalization			0.029
No hospitalization	25 (5.29%)	448 (94.71%)	
Hospitalization for 1-5 days	3 (6.12%)	46 (93.88%)	
Hospitalization for more than five days	12 (12.63%)	83 (87.37%)	

## Discussion

The World Health Organization declared the end of COVID-19 as a global health emergency on March 5, 2023, three years after its initial emergence [15]. Despite global efforts to move beyond the acute phase, people still suffer from a multisystem debilitating condition after acute infection, which persists despite recovery [16]. In the present cohort of 617 participants, 40 (6.48%) participants were still experiencing persistent symptoms at follow-up. Compared to earlier studies from India (7.1%-47.17%) [17-21] and globally (10%-63%) [22-25], our study had a lower prevalence of PCC. Similarly, PCC was more prevalent among those hospitalized (10.42%) than those home-isolated (5.28%). This finding is concurrent with global studies where the prevalence among hospitalized cases (50%-70%) was higher than the non-hospitalized cases (10%-30%) [22,24]. The follow-up period after the acute infection also significantly influences PCC prevalence. Indian studies showed that the PCC prevalence declined from 12.1% and 33.2% at four weeks to 9.9% and 12.8% at 12 weeks, respectively [18,20]. Similarly, a systematic review article reported a similar trend of declining prevalence from 63.2% at four weeks to 45.9% at 12 weeks [25]. Therefore, several factors likely contributed to the observed variations in PCC prevalence, including the selection of the study participants (asymptomatic/symptomatic, hospitalized/non-hospitalized, or vaccinated/non-vaccinated), differing follow-up periods, and the timing of the study (pre-Delta, Delta, or Omicron waves).

Notably, most prevalence studies were conducted between 2020 and 2021, when cases were primarily due to the index SARS-CoV-2 virus or the Delta variant and its sub-lineages. The present study, however, includes participants from both Delta and Omicron waves. Notably, the occurrence of PCC was significantly higher in those infected during the Delta wave (12.38%) than those infected during the Omicron wave (5.27%). Our findings are consistent with the UK's Office for National Statistics study, where long COVID was 50% less common in participants with Omicron variant infection than those with Delta variant infection [26]. This discrepancy in prevalence may be mainly due to the greater severity of illness, higher rates of hospitalization, and more debilitating symptoms associated with the Delta variant compared to the Omicron variant [27]. Prior research shows that the Delta variant has increased lung infectivity and fusogenicity, increasing its pathogenicity [28]. Additionally, compared to Omicron cases, Delta-infected individuals experienced more pronounced symptoms such as loss of smell and taste [27], attributed to altered function of sensory neurons and death of supporting cells due to inflammation [29]. Therefore, it is likely that tissue injury resulting from abnormal inflammatory reactions during acute infection played a significant role in the development of persistent symptoms in Delta-infected cases.

The most common long COVID symptom reported in the present study was malaise (25%), followed by shortness of breath (20%), fatigue (17.5%), joint pain (15%), and frequent episodes of cough and cold (15%). The global pooled prevalence of specific symptoms shows that fatigue (0.23, 95% CI: 0.17-0.30) is the most prevalent symptom, followed by memory problems (0.14, 95% CI: 0.10-0.19), dyspnea (0.13, 95% CI: 0.11-0.15), sleep disorders (0.11, 95% CI: 0.05-0.23), and joint pain (0.10, 95% CI: 0.04-0.22) [24]. Likewise, in Indian studies, the prevalence of post-COVID symptoms has been reported with variations, with fatigue, cough, dyspnea, sleep disturbances, body ache, and myalgia being the most common [18-21]. Also, 1.94% of participants in the present study had developed a new condition after COVID-19 infection. In this study, cases with more than four symptoms during acute infection, moderate to severe disease, those who required hospitalization, and those hospitalized for more than five days showed a statistically significant association with post-COVID-19 symptoms. Our findings are consistent with other Indian studies where more than five symptoms, severe disease, and hospital admission were significantly associated with long COVID [18,19,21]. In contrast to other studies, the present study did not find a significant association of long COVID with female gender, older age group, presence or absence of comorbidities, and vaccination status at the time of acute infection. The variation in vaccination status between the groups due to amendments in the vaccination policy across different waves adds further complexity. Various studies highlight varying perspectives on the relationship between vaccination and the development of long COVID symptoms. While one study suggests no significant difference in the development of long COVID symptoms between vaccinated and unvaccinated individuals, others indicate that vaccines offer partial protection, with the risk of long COVID [22] reducing from 41.8% in unvaccinated individuals to 16% after three-dose vaccination [30]. Similarly, in a study from India, the odds of developing PCC following two doses of vaccination were reduced by 45% compared to unvaccinated individuals [20]. Therefore, the impact of vaccination on the incidence of long COVID remains a subject that requires further research.

A wide range of biological factors have been hypothesized to contribute to the development of post-COVID-19 symptoms. Factors such as residual tissue injury due to prolonged hospital/ICU stay and mechanical ventilation, autoimmunity due to molecular mimicry between pathogen and host proteins, and the development of vascular clots leading to decreased pulmonary and cardiac perfusion have been responsible for persistent respiratory and cardiovascular symptoms such as dyspnea, chest pain, palpitations, tachycardia, and arrhythmias. The possible mechanisms for neurological and cognitive dysfunctions such as memory loss, brain fog, sleeping disorder, loss of taste and smell, and autonomic dysfunction include neuroinflammation, micro-clots, and endothelial dysfunction leading to decreased brain perfusion and viral persistence in brain tissues that may result in persistent symptoms through direct viral cytopathic effects, dysregulated immune response leading to a prolonged state of inflammation, and dysfunctional brain stem signaling. Similarly, it has been hypothesized that infection-induced widespread inflammation can potentially lead to gut microbiome dysbiosis. This, in turn, may stimulate the regional nerves, leading to persistent abnormal gastrointestinal symptoms [30,31]. Moreover, it is crucial to understand and identify human genome variants that may play a role in modulating their response to environmental insults such as SARS-CoV-2 infection, which can lead to PCC development [31].

At the proteomics level, a study investigating the plasma proteome of long COVID cases revealed that there is a differential protein expression that disrupts tissue recovery mechanisms, correlating with the coordinated actions of hypoxia-inducible factor (HIF), tumor necrosis factor-α (TNF-α), and vascular endothelial growth factor A (VEGFA) signaling, all linked to angiopoietin (ANGPT1). This disruption leads to persistent and diffuse symptoms even after recovery. Notably, elevated levels of APP antigen in the plasma of long COVID patients suggest that peptides from APP gene products may form cytotoxic aggregates, potentially contributing to long-term neurological symptoms. Furthermore, the study highlights that the alterations in integrin signaling and an increased presence of the inflammatory molecule CCL5 in the plasma of long COVID cases lead to a state of prolonged inflammation. Dysregulated inflammation, combined with improper healing and pathological remodeling of cardiac tissue, may be responsible for the observed cardiac dysfunction in long COVID cases [32]. Hence, as evident, the underlying mechanisms of long COVID are complex and multifaceted, necessitating continued research efforts to fully understand its pathogenesis.

Despite the advantages of long-term follow-up duration and comparison of PCC prevalence among Delta and Omicron variant-infected cases, the study has few limitations. The study relies on the self-reported symptoms obtained from telephonic interviews that could introduce recall bias. Additional information on clinical and laboratory results correlating with the symptom findings could not be collected. Furthermore, due to the exclusion of individuals below 18 years of age, the present study findings may not be generalized to the pediatric population. Therefore, more comprehensive and large-scale cohort studies, including children and adolescents, are essential to assess, monitor, and understand the symptoms and pathogenesis of long COVID.

## Conclusions

Long COVID, extending beyond the acute phase of COVID-19, results in prolonged disability and illness. This preliminary study provides critical insights into the burden and characteristics of post-COVID-19 conditions among SARS-CoV-2 variant-identified cases in Maharashtra, India. The study shows that despite the higher infectivity of the Omicron variant, the incidence of PCC among those infected with the Omicron variant was relatively lower than those infected with the Delta variant. In this study, there is a significant association between disease severity, hospitalization during acute illness, and hospitalization for more than five days with PCC. The chronic nature and the widespread occurrence of its consequences suggest that the effects of long COVID will have social, economic, and political impact even after the pandemic subsides. Therefore, the current study serves as a step toward understanding the potential burden of PCC on healthcare resources that will be instrumental in guiding future research and healthcare strategies to manage these conditions in Maharashtra and beyond.

## References

[1] Mizrahi B, Sudry T, Flaks-Manov N (2023). Long covid outcomes at one year after mild SARS-CoV-2 infection: nationwide cohort study. BMJ.

[2] (2023). A clinical case definition of post COVID-19 condition by a Delphi consensus. https://www.who.int/publications/i/item/WHO-2019-nCoV-Post_COVID-19_condition-Clinical_case_definition-2021.1.

[3] (2023). Post COVID-19 condition (long COVID). https://www.who.int/europe/news-room/fact-sheets/item/post-covid-19-condition.

[4] (2021). Long COVID. https://www.covid.gov/Longcovid/definitions.

[5] (2021). Long COVID or post-COVID conditions. https://www.cdc.gov/coronavirus/2019-ncov/Long-term-effects/index.html.

[6] (2021). National comprehensive guidelines for management of post-COVID sequelae. https://www.mohfw.gov.in/pdf/NationalComprehensiveGuidelinesforManagementofPostCovidSequelae.pdf.

[7] (2021). #IndiaFightsCorona COVID-19. https://www.mygov.in/covid-19/.

[8] Das R, Karyakarte R, Joshi S (2021). Clinical characteristics of AY.4 infections are similar to B.1.617.2 infections: a preliminary study. Indian J Bas App Med Res.

[9] Karyakarte RP, Das R, Taji N (2022). An early and preliminary assessment of the clinical severity of the emerging SARS-CoV-2 Omicron variants in Maharashtra, India. Cureus.

[10] Karyakarte RP, Das R, Dudhate S (2023). Clinical characteristics and outcomes of laboratory-confirmed SARS-CoV-2 cases infected with Omicron subvariants and the XBB recombinant variant. Cureus.

[11] (2023). Global COVID-19 clinical platform case report form (CRF) for post COVID condition (post COVID-19 CRF). https://www.who.int/publications/i/item/global-covid-19-clinical-platform-case-report-form-(crf)-for-post-covid-conditions-(post-covid-19-crf-).

[12] (2023). Guidelines for COVID-19 vaccination of children between 12-14 years of age. https://vikaspedia.in/health/health-campaigns/all-about-covid-vaccines/guidelines-for-covid19-vaccination-of-children-between-1214-years-of-age.

[13] (2023). Revised guidelines for home isolation of mild/asymptomatic COVID-19 cases. https://covid19.india.gov.in/document/revised-guidelines-for-home-isolation-of-mild-asymptomatic-covid-19-cases/.

[14] (2023). Government of India, Ministry of Health & Family Welfare: Revised guidelines for home isolation of mild/asymptomatic COVID-19 cases. https://www.mohfw.gov.in/pdf/RevisedHomeIsolationGuidelines05012022.pdf.

[15] (2023). Statement on the fifteenth meeting of the IHR (2005) Emergency Committee on the COVID-19 pandemic. https://www.who.int/news/item/05-05-2023-statement-on-the-fifteenth-meeting-of-the-international-health-regulations-(2005)-emergency-committee-regarding-the-coronavirus-disease-(covid-19)-pandemic?gclid=Cj0KCQjwwvilBhCFARIsADvYi7LuVI5QzjCI25N8_P5iC1bwQH3Nr_oAuqHkQys222WrDftu6HKA5xcaAhopEALw_wcB.

[16] The Lancet (2023). Long COVID: 3 years in. Lancet.

[17] Anjana NK, Annie TT, Siba S, Meenu MS, Chintha S, Anish TS (2021). Manifestations and risk factors of post COVID syndrome among COVID-19 patients presented with minimal symptoms - a study from Kerala, India. J Family Med Prim Care.

[18] Naik S, Haldar SN, Soneja M (2021). Post COVID-19 sequelae: a prospective observational study from Northern India. Drug Discov Ther.

[19] Arjun MC, Singh AK, Pal D (2022). Characteristics and predictors of long COVID among diagnosed cases of COVID-19. PLoS One.

[20] Senjam SS, Balhara YP, Kumar P (2021). Assessment of post COVID-19 health problems and its determinants in North India: a descriptive cross section study. medRxiv.

[21] Chopra N, Chowdhury M, Singh AK (2021). Clinical predictors of long COVID-19 and phenotypes of mild COVID-19 at a tertiary care centre in India. Drug Discov Ther.

[22] Davis HE, McCorkell L, Vogel JM, Topol EJ (2023). Long COVID: major findings, mechanisms and recommendations. Nat Rev Microbiol.

[23] Duggal P, Penson T, Manley HN (2022). Post-sequelae symptoms and comorbidities after COVID-19. J Med Virol.

[24] Chen C, Haupert SR, Zimmermann L, Shi X, Fritsche LG, Mukherjee B (2022). Global prevalence of post-coronavirus disease 2019 (COVID-19) condition or long COVID: a meta-analysis and systematic review. J Infect Dis.

[25] Fernández-de-Las-Peñas C, Palacios-Ceña D, Gómez-Mayordomo V, Florencio LL, Cuadrado ML, Plaza-Manzano G, Navarro-Santana M (2021). Prevalence of post-COVID-19 symptoms in hospitalized and non-hospitalized COVID-19 survivors: a systematic review and meta-analysis. Eur J Intern Med.

[26] (2023). Self-reported long COVID after infection with the Omicron variant in the UK: 18 July 2022. https://www.ons.gov.uk/peoplepopulationandcommunity/healthandsocialcare/conditionsanddiseases/bulletins/selfreportedLongcovidafterinfectionwiththeomicronvariant/18july2022.

[27] Menni C, Valdes AM, Polidori L (2022). Symptom prevalence, duration, and risk of hospital admission in individuals infected with SARS-CoV-2 during periods of omicron and delta variant dominance: a prospective observational study from the ZOE COVID Study. Lancet.

[28] Meng B, Abdullahi A, Ferreira IA (2022). Altered TMPRSS2 usage by SARS-CoV-2 Omicron impacts infectivity and fusogenicity. Nature.

[29] Mastrangelo A, Bonato M, Cinque P (2021). Smell and taste disorders in COVID-19: from pathogenesis to clinical features and outcomes. Neurosci Lett.

[30] Perumal R, Shunmugam L, Naidoo K (2023). Long COVID: a review and proposed visualization of the complexity of long COVID. Front Immunol.

[31] Proal AD, VanElzakker MB (2021). Long Covid or post-acute sequelae of COVID-19 (PASC): an overview of biological factors that may contribute to persistent symptoms. Front Microbiol.

[32] Iosef C, Knauer MJ, Nicholson M (2023). Plasma proteome of long-COVID patients indicates HIF-mediated vasculo-proliferative disease with impact on brain and heart function. J Transl Med.

